# Sir William MacCormac

**Published:** 1902-01

**Authors:** 


### OBITUARY.

Sir William MacCormac, Bart., president of the Royal College of Surgeons, died suddenly from heart disease at Bath, December 4, 1901. He was born in Belfast in 1836, his father, Dr. Henry MacCormac, also being a physician. He received his education in Belfast, Dublin, and Paris. After making a reputation as a surgeon in the hospitals of the United Kingdom and winning the gold medal of the Queen's University he saw service at Metz and Sedan in the Franco-German War as Surgeon-in-Chief of the Anglo-American ambulance, and also during the Turko-Servian War, in 1876. For twenty years he was one of the senior surgeons and lecturer at St. Thomas's Hospital, as well as consulting surgeon and examiner in many other institutions.

In consideration of his services as secretary general of the International Medical Congress, held in London in 1881, he was knighted by the Queen. In 1897, at the Queen's Jubilee, he was made a baronet and appointed surgeon-in-ordinary to the Prince of Wales, whom he attended when suffering from the effects of an accident. He also has been honored by many foreign governments. When the Boer war broke out he went with the British Army to Africa as consulting civil surgeon, and was present during the early fighting in Natal. He was the author of a great number of treatises on various subjects, most of which were contributed to different medical journals or societies. Among these may be mentioned: Surgical operations, Antiseptic surgery, Notes and recollections of an ambulance surgeon. In 1899 he delivered the Hunterian oration before the Royal College of Surgeons.

